# Rivalries for attention: insights from a realist evaluation of a postgraduate competency-based medical education implementation in Canada

**DOI:** 10.1186/s12909-022-03661-8

**Published:** 2022-07-29

**Authors:** Christen Rachul, Benjamin Collins, Ming-Ka Chan, Ganesh Srinivasan, Joanne Hamilton

**Affiliations:** 1 Office of Innovation and Scholarship in Medical Education, Max Rady College of Medicine, University of Manitoba, S204, Medical Services Building, 750 Bannatyne Ave, Winnipeg, MB R3E 0W2 Canada; 2 Department of Psychiatry, Max Rady College of Medicine, University of Manitoba, Winnipeg, Canada; 3 Department of Anthropology, University of Manitoba, Winnipeg, Canada; 4 Department of Pediatrics and Child Health, Max Rady College of Medicine, University of Manitoba, Winnipeg, Canada

**Keywords:** Post graduate medical education, Competency-based medical education, Realist evaluation, Specialty training, Curriculum Management System, Implementation

## Abstract

**Background:**

Implementing competency-based medical education (CBME) in post-graduate medical education (PGME) is a complex process that requires multiple systemic changes in a complex system that is simultaneously engaged in multiple initiatives. These initiatives often compete for attention during the implementation of CBME and produce unintended and unanticipated consequences. Understanding the impact of this context is necessary for evaluating the effectiveness of CBME. The purpose of the study was to identify factors, such as contexts and processes, that contribute to the implementation of CBME.

**Methods:**

We conducted a realist evaluation using data collected from 15 programs through focus groups with residents (2 groups, *n* = 16) and faculty (one group, *n* = 8), and semi-structured interviews with program directors (*n* = 18), and program administrators (*n* = 12) from 2018 to 2021. Data were analyzed using a template analysis based on a coding framework that was developed from a sample of transcripts, the context-mechanism-outcomes framework for realist evaluations, and the core components of CBME.

**Results:**

The findings demonstrate that simultaneous initiatives in the academic health sciences system creates a key context for CBME implementation – rivalries for attention – and specifically, the introduction of curricular management systems (CMS) concurrent to, but separate from, the implementation of CBME. This context influenced participants’ participation, communication, and adaptation during CBME implementation, which led to change fatigue and unmet expectations for the collection and use of assessment data.

**Conclusions:**

Rival initiatives, such as the concurrent implementation of a new CMS, can have an impact on how programs implement CBME and greatly affect the outcomes of CBME. Mitigating the effects of rivals for attention with flexibility, clear communication, and training can facilitate effective implementation of CBME.

**Supplementary Information:**

The online version contains supplementary material available at 10.1186/s12909-022-03661-8.

## Background

In 2017, specialty training programs in Canada began a staged implementation of a hybrid version of competency-based medical education (CBME) called Competence by Design (CBD) [1] that blends the core components of CBME [2] with aspects of time-based approaches. CBME implementation requires multiple changes and adaptations within complex academic health sciences systems [1, 3–7]. These complex systems are simultaneously engaged in a wide range of activities and initiatives that potentially interact with and influence the implementation of CBME by competing for attention, time, and resources. Pawson describes these simultaneous initiatives as rivalries [8]. This context for CBME implementation can have a profound effect on outcomes [4, 9] and is a necessary consideration in evaluating its effectiveness [10, 11].

Implementation evaluations provide valuable insight into how contexts and processes contribute to outcomes [11], but many of the evaluation studies that have focused on the implementation of CBME have focused largely on processes with minimal consideration for context, particularly the effect of simultaneous activities and initiatives [5, 12–14]. As CBME gains traction internationally, it is important to gather more evidence for how context influences CBME outcomes in order to facilitate more effective implementation and to generate more robust evidence for CBME. As such, we conducted a realist evaluation [8, 15, 16] of the implementation of CBD in postgraduate medical education (PGME) at the University of Manitoba (UM), Canada that considered the geographical, institutional, and disciplinary contexts at UM. The objectives of the study were to identify factors (i.e., contexts and processes) that contribute to the implementation of CBME at UM. The large-scale study uncovered several contexts that impacted the UM implementation of CBD, and in this paper, we focus on one crucial context that affected implementation: simultaneous activities and initiatives, or rivalries.

## Methods

We conducted a realist evaluation of the implementation of CBD from 2018 to 2021. The study was approved by the UM Health Research Ethics Board (REB Study H2017:313). All methods were carried out in accordance with relevant guidelines and regulations.

### Realist evaluation

Realist evaluations start with the assumption that it is not an intervention that works, but the participants’ engagement with an intervention that produces particular outcomes. The focus is on what works for whom, under which circumstances, and why [8, 15, 16]. Realist approaches begin with an initial program theory that provides a broad-scale hypothesis about how and why a program produces particular outcomes. While the use of program theory and logic models are common in program evaluation, (e.g. [12],) in realist evaluation, the program theory is refined through iterative testing and analysis that uncovers “context + mechanism = outcome” (CMO) configurations that demonstrate how particular circumstances (C) and the actions and reactions of participants (M) lead to particular outcomes (O) [8, 15]. This approach considers variability between intervention sites and stakeholders, and therefore is of merit for understanding the nature of the implementation of CBME in PGME [16]. Our initial program theory was informed by the core components of CBME [2] and implementation planners [17]. Thus, our initial program theory described how, with all programs being treated equal (C), a successful CBME implementation can be facilitated by the readiness checklists (M) and measured by the presence of CBME’s core components (O) (Table 1).

**Table 1:** Initial Program Theory for CBME as it relates to rivalries for attention [2, 18]

Context (C)
Specialty training programs
** + Mechanisms (M)**
1. Team and Resources a. Learning about CBD using a variety of resources b. Develop a team and connect with other people in your institution to learn about policies and practices c. Develop a QI plan 2. Capacity Building a. Pilot entrustable professional activities (EPAs) b. Conduct faculty and resident development c. Promote a culture of feedback and coaching d. Develop resident-friendly resources
** = Outcomes (O)**
1. Outcome competencies are clearly articulated 2. Competencies and their developmental markers are sequenced progressively 3. Learning experiences facilitate the developmental acquisition of competencies 4. Teaching practices promote the developmental acquisition of competencies 5. Assessment practices support & document the developmental acquisition of competencies

### Participants and setting

All UM specialty training programs who implemented CBME from 2017–2020 were invited to participate in the realist evaluation. Fifteen of the eligible 32 programs agreed to participate. Participants were recruited from these programs and included residents, faculty members, program directors (PD), and program administrators (PA).

### Data collection

Data were collected during three realist evaluation phases: 1) theory gleaning (2018–2019); 2) theory refining (2020); and 3) theory consolidation (2021) in order to test and refine our program theory [18]. During each phase of the study, CR and a research assistant conducted focus groups with residents (two groups, *n* = 16) and faculty members (one group, *n* = 8), and semi-structured interviews with PDs (*n* = 18 with 11 different PDs), and PAs (*n* = 12 with 6 different PAs). The research team developed and revised the interview and focus group guides to reflect the goals of each realist evaluation phase including the specific contexts, mechanisms, and outcomes we explored during each phase (see Additional file 1). Interviews and focus groups were audio-recorded and transcribed verbatim.

### Data analysis

We conducted a template analysis of the interviews and focus groups [19]. During phase 1, CR and a research assistant conducted initial coding of a sample of transcripts. The research team then developed a coding framework based on the initial coding of transcripts, realist evaluation’s CMO framework [8, 15], and our initial program theory [2, 17]. CR applied the coding framework to all phase 1 data to glean preliminary CMO findings. During phase 2, BC applied and refined the coding framework to all of the phase 2 data in discussion with the research team as new concepts and patterns were identified. This revised coding framework was then used to re-analyze all phase 1 data. Finally, CR applied the finalized coding framework to the phase 3 data to consolidate our program theory. The research team met at regular intervals to discuss and refine the findings.

### Reflexivity

The research team has a variety of experience and expertise that affected how we collected, analyzed, and interpreted our data. CR, BC, and JH are PhD-trained researchers who brought experience with conducting qualitative research and program evaluation in medical education to data collection and analysis. Additionally, CR and JH are involved in the CBME steering committee in which CR leads a CBME evaluation working group and JH offers educational support to programs. MKC and GS are educational leaders and clinicians in programs that had begun preparation but not yet launched CBME during the data collection period. They brought their experience with implementing educational innovations, facilitating faculty development, and teaching in specialty training programs to the interpretation of data.

## Results

Through our iterative analysis of interviews and focus groups, we identified how the implementation of CBD was affected by multiple concurrent initiatives, which rivaled for the attention of residents, faculty members, and staff. These concurrent initiatives included new PGME accreditation standards, organizational changes in the healthcare system, and from 2020 onward, changes associated with the COVID-19 pandemic; however, the biggest rival initiative was the concurrent implementation of two new curriculum management systems (CMS) early on in the staged implementation of CBD. Participants consistently identified the new CMS and described mechanisms and outcomes that directly impacted the implementation of CBME.

### Context

The UM’s first cohort to launch CBME also implemented a new CMS, ePortfolio [20], at the same time. About one year later, just prior to the second cohort’s launch of CBME, another CMS, Entrada (officially known as Elentra [21]), was introduced across the multidisciplinary faculty of health sciences. The introduction of Entrada was separate from the implementation of CBME, although the specialty training programs were required to switch from ePortfolio to Entrada. Decisions regarding the CMS, for example, access and components, addressed needs for the whole faculty of health sciences and not just CBME. Discussion regarding the implementation of these two new CMS arose during every interview and focus group in phase 1 and was subsequently selected as a context to explore further in phase 2 and test in phase 3. The decision to launch two new CMS early on in the staged implementation presented a *rivalry for attention* that affected the implementation of CBME in multiple ways (Table 2). One was the timing of the announcement of the decision that gave little preparation time prior to the launch of CBME in the second cohort of programs and required residents, faculty members, and staff from the first cohort to learn two new programs within a year of each other. In addition, some of the key CMS components required to effectively facilitate CBME, such as tracking progress and generating reports for competence committees, were still being developed during the first few years that CBME was being implemented. While changes to the CMS were ongoing, these changes were not always communicated to the people most affected and the CMS training sessions and videos were not always adequate. Finally, a faculty-wide decision was made that required users to access the CMS with their university e-mail addresses, which uncovered additional issues around university appointments for teachers and issues of compatibility with technology across the healthcare system.

**Table 2:** Example quotes for Context, Mechanisms, and Outcomes

Context	“I think a lot of the problems were that nobody knew what software platform we were going to use. And then a month beforehand it switched. So, I think there was a lot of problems with the roll-out” (PD4_2019) “The whole ePortfolio thing has not helped. We're getting ready for one system, now you've got another system, and at the same time we're introducing other clinical systems.” (F1_2019) “I do think that it should have probably been planned a little bit more carefully, because we're rolling out a system that doesn't have all the components in place. There's [sic] new versions coming out all the time” (PA6_2020)
Mechanisms	**Participation** “The fact that the university is using just the University of Manitoba email addresses. Well, I'd say 95% of our attendings don't routinely use their University of Manitoba email addresses and I'd say 50% or 60% don't even know how to access them and that is with several dedicated sessions on how to access your University of Manitoba email” (PD6_2020) “No one is logging into this thing. The app doesn't work, and the website is not conducive to data collection, so you're not going to get a bunch of 60-year-old surgeons pulling out their phone.” (PD11_2021) **Communication** “I need to know how Entrada works because that's just the main communication tool of getting the EPAs completed, and that's essentially where I've been having my most difficulty is understanding how Entrada works because it's not the most straightforward program I'm learning” (PA1_2020) “[Faculty development] was mainly about making sure that they could actually get in Entrada, and have their U of M account working. The hardest part was going to them and saying, “This is how you claim your ID. This is how you do this.” (PA2_2020) **Adaptation** “Most of us have actually created a separate spreadsheet or an Excel document of some sort where you have to say, okay I need this, and then you need to get it from, oh, I need this one from a nurse and an anesthetist and then someone else.” (R1_2020) “I would say the most difficult part or one challenge I guess is just making sure the [faculty] appointments are all set up on [Entrada] because if they're not then […] the resident has to print it, the EPA, and then they have to get it filled out and then I have to input it.” (PA3_2020)
Outcomes	**Change Fatigue** “Entrada is probably the biggest difficulty. It just was a lot of change at once. And I'm fine with change, but not everyone loves change. So, when we are doing different forms, different ways to teach, now we're using a different system [...] There's a lot of changes at once, because everything was July 1st for us. Like with Entrada, competency, all of it, was July 1st for us.” (PA6_2020) The Family Medicine curriculum “sounds super similar and it's on Entrada. Now [faculty] think everything on Entrada has got to be CBD. It's not.” (PA3_2020) They used the UM email, which most staff frankly don't use,...is another barrier. It just adds another layer and more workarounds and more education... but if nobody's using it, it's just been more work. (PD3_2021) **Unmet Expectations** “all of the effort that went into showing [faculty members] what it was, then three months go by and they haven't done a single evaluation, then they probably forgot what they were supposed to do as well. So then when it did start happening, there was a lot of, “Well, I don't know how to do this. I don't have an account.” My residents are scrambling trying to get it done before their deadline, but faculty didn't know how to do it.” (PD4_2019) “I think we've had a lot of issues with staff not having accounts set up or […] different emails or these little things that really aren't a big deal, but have been a big barrier to getting evaluations completed.” (PD7_2020) “Some don't know their log-in info, for instance, and can't do [the assessment] on the spot and then it doesn't get done or doesn't get done until weeks later, which then isn't the point of all of this in the first place, in which case it's really not adding anything to anyone's learning. So that's the issue with Entrada.” (R1_2020) **Earlier Problem Identification** “I would say that we have a better sense of where our residents are at any given time and can quickly move towards meeting with our residents if there's some difficulty, identify and address it quickly.” (PD2_2020) “It's easier as a program director to see where a resident stands amongst their peers, and if they are struggling or not meeting certain components, it's easier to identify those and to try to act on them early on.” (PD7_2020)

The interviews were cataloged by position (PD, PA, F = Faculty, and R = Resident), program (numbered), and year, so PD4_2019 indicates an interview with the PD from program 4 that took place in 2019

### Mechanisms

We identified three key mechanisms, or actions and reactions, in response to the concurrent implementation of new CMS: participation, communication, and adaptation (Table 2). During phase 1, we identified several smaller mechanisms that, during phase 2, were tested for their existence across programs and their effect on the outcomes. Subsequently additional mechanisms were identified and grouped together into these 3 key mechanisms that were finalized during phase 3. Challenges with the implementation of new CMS affected faculty members’ and residents’ *participation* in new CBME practices. For example, some programs reported that some faculty members did not know their university e-mail addresses so were unable to register and login to the new CMS and, as a result, were not completing assessments. The impact was greatest during the first two years of implementation when there were two rapid changes in CMS, the current CMS requires a university e-mail address to register, and other technical limitations or poor communication of changes to the ongoing development of the CMS. Since the current CMS is a faculty-wide initiative, common challenges associated with learning a new software program decreased in the following years as all residents, faculty members, and administrators gained more experience with the CMS even prior to launching CBME. However, the software has continued to change and develop and access issues remain, continuing to impact faculty members’ and residents’ use of the CMS to engage in CBME.

In addition, *communication* about the implementation of CBME was dominated by topics related to the new CMS. In particular, faculty, staff, and resident development initiatives focused on how to gain access to and use the CMS, turning people’s attention away from other important CBME-related topics. PDs and PAs reported providing large group sessions on the CMS in preparation before the launch of CBME and then following up individually with faculty members over the first several months post-launch. Following the first year of implementation of the current CMS, the focus on CMS training in preparation for CBME lessened for programs whose faculty members gained experience using the system with non-CBME residents and with residents from other CBME programs. However, the focus on CMS training remained for programs with small numbers of residents and who do not often host off-service residents.

Finally, *adaptation* became an important and common approach to navigating the limitations of a developing CMS. In response to faculty members’ challenges with accessing the CMS and to reduce other barriers to its use, some residents initially began printing off assessments for faculty to complete and for PAs to upload manually. This approach was further adapted when residents began bringing mobile devices to encourage faculty members to complete assessments immediately following an observation. Additionally, the CMS initially lacked easy methods for tracking completion of assessments and for developing useful reports on resident progress for competence committees. Many individuals and programs developed additional methods for tracking and reporting progress outside of the CMS to fill in this gap.

### Outcomes

The concurrent implementation of a new CMS and resulting mechanisms contributed to the outcomes of the implementation of CBME (Table 2). Several preliminary outcomes were identified during phase 1 and, during phase 2, were further explored and narrowed to the outcomes that persisted through each study phase. First, PDs, PAs, and faculty members, particularly those involved in programs from the first two cohorts to implement CBME, experienced *change fatigue* due to the concurrent implementation of two new CMS and CBME. Change fatigue was further exacerbated by the increased workloads brought on by learning a new CMS, training others to use the CMS, and developing workarounds to address the limitations of the CMS. This focus on the new CMS, particularly for the first two cohorts to implement CBME, often led to the conflation of the CMS with CBME.

Second, as a result of the ways that people reacted to the concurrent implementation of a new CMS, programs reported *unmet expectations* for the collection and use of CBME assessment data. Programs reported that they were not collecting as much assessment data as expected, both in terms of quantity and quality. This deficiency was largely due to faculty members’ delayed interactions with the CMS either because of challenges with accessing the CMS or limitations for tracking completed assessments. As a result of delayed or incomplete assessments, residents’ engagement in CBME practices, particularly their ability to learn *from* assessment, impeded their learning experience. However, even though the expectations for the quantity and quality of resident data had not been met, programs reported that the data they do collect has helped the competence committees to earlier identification of problems with resident performance sooner than in the previous time-based model.

**Fig. 1:**
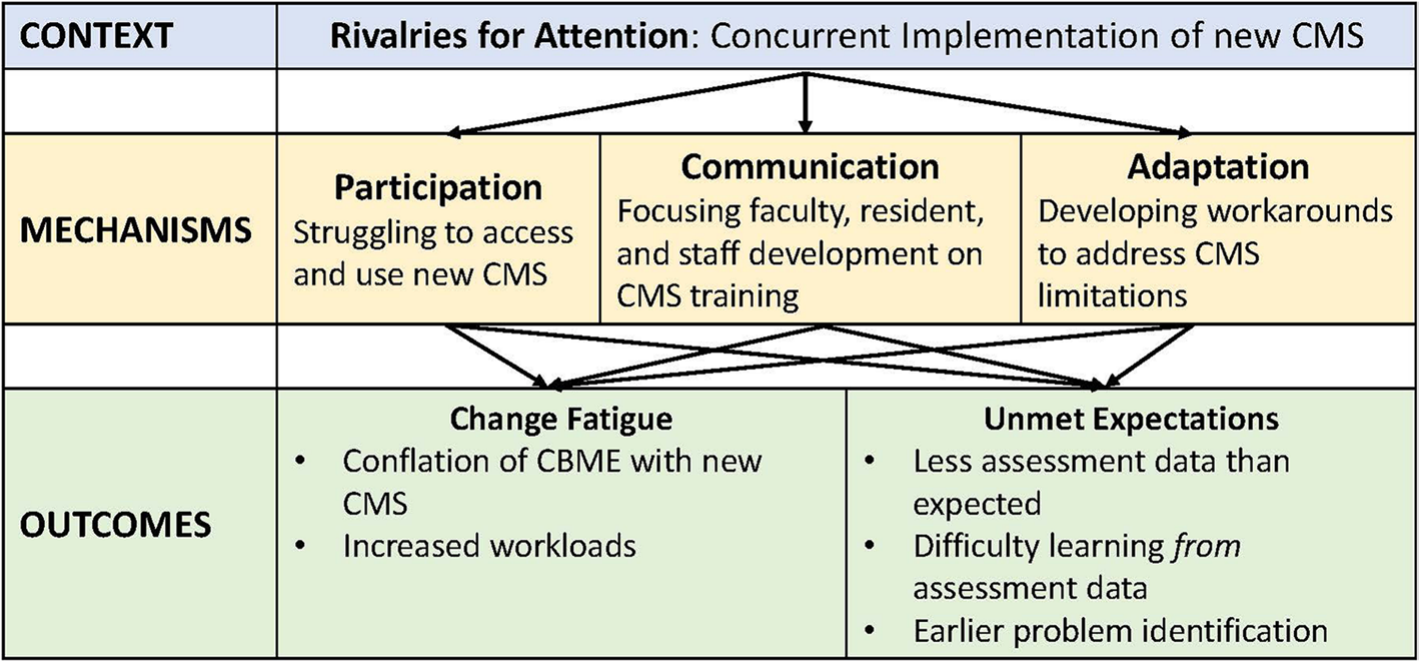
Description of the CMOs related to the concurrent implementation of CBME and a new CMS

Some outcomes of the implementation of CBME, namely change fatigue and unmet expectations, were heavily influenced by the context of multiple concurrent initiatives that rivaled for participants attention, primarily the concurrent implementation of two new CMS, that generated the mechanisms of participation, communication and adaptation. Further, we found that the mechanisms and outcomes related to rivalries for attention are interconnected and do not lend themselves to discrete CMO configurations as depicted in Fig. 1.

## Discussion

The findings demonstrate how a simultaneous activities and initiatives can rival for the attention of residents, faculty members, and staff when implementing CBME, and in particular, how a new CMS can rival for people’s attention when implemented concurrently with CBME. Our initial program theory focused on the contexts and mechanisms within the specialty training programs, but our analysis suggests that some of the outcomes of the implementation of CBME are influenced by the contexts of the academic health sciences system. This context includes the decisions that affect CBME implementation but are not exclusive to CBME, such as the concurrent implementation of a new CMS. This concurrent implementation affected how people participated in new CBME practices, changed communication strategies as programs prepared for and launched CBME, and prompted adaptations to mitigate limitations of the CMS and facilitate CBME practices. Residents, faculty members, and staff experienced change fatigue as they navigated multiple new ways of working. Additionally, expectations for CBME to improve the information about resident progress and the learning experience were challenged by ongoing changes and limitations of the new CMS.

The influential role of CMS on CBME has also been identified in previous research. CMS are a key factor in the success of CBME, particularly for completing, tracking and reporting assessment data [5, 13, 22–24]. Yet, previous research has shown that the implementation of new CMS for CBME can be resource-intensive [13] and can create frustration with the new interface and increase the amount of time required to conduct assessments [5, 25]. We build on this research by demonstrating how the concurrent implementation of a new CMS can compound the challenges with and distract from the effective implementation of CBME and potentially have an impact on the outcomes of CBME. While challenges with the CMS limited the collection and reporting of assessment data and residents’ ability to learn from this data, programs still reported that the data they did collect allowed them to identify concerns with resident progress sooner, which is a key component of CBME [2, 3]. This finding suggests that, by reducing the effects of the implementation of a new CMS, CBME can produce rich and valuable information for and about residents.

There are a number of ways to mitigate the challenges imposed by implementing a new CMS for CBME. First, developing a timeline for implementing the new CMS that allows ample time for training and piloting the new CMS prior to the launch of CBME can reduce potential change fatigue, identify its limitations in low stakes circumstances, and clarify the boundaries between the CMS and CBME. Second, developing a clear communication strategy for changes to the CMS and comprehensive CMS training and technical support that is separate, yet complementary to CBME-specific faculty, resident, and staff development initiatives can help alleviate the burden on PDs and PAs to support teachers and residents as they navigate the new practices associated with CBME[5, 23, 24].Finally, given the large impact that CMS can have on the implementation of CBME, more research is required on how users interact with CMS and what interface and tools are required for the CMS to facilitate CBME rather than rival for its attention. We have incorporated these suggestions into our revised program theory that added mechanisms related to the institutional level (Table 3).

**Table 3:** Revised Program Theory for CBME as it relates to rivalries for attention [2, 17]

Context (C)
Rivalries for attention
** + Mechanisms (M)**
1. Team and Resources *Program-level* a. Learning about CBD using a variety of resources and faculty development strategies b. Develop a team and connect with other people in your institution to learn about policies and practices *Institutional-level* c. Provide human and financial resources for additional activities related to CBME such as protected time or additional administrative resources d. Develop training resources and technical support for CMS and other technology that supports CBME 2. Capacity Building *Program-level* a. Pilot EPAs b. Conduct faculty and resident development c. Promote a culture of feedback and coaching d. Develop resident-friendly resources *Institutional-level* e. Develop a CMS that supports and facilitates the curricular and assessment needs of CBME f. Stagger implementation of new initiatives when possible
** = Outcomes (O)**
1. Outcome competencies are clearly articulated 2. Competencies and their developmental markers are sequenced progressively 3. Learning experiences facilitate the developmental acquisition of competencies 4. Teaching practices promote the developmental acquisition of competencies 5. Assessment practices support & document the developmental acquisition of competencies

More broadly, while CBME is intended to provide greater standardization in medical training and social accountability, embracing flexibility and contextualization during implementation can help programs navigate the challenges associated with concurrent initiatives that are expected, such as changes to accreditation standards, and unexpected, such as the COVID-19 pandemic [4]. Additionally, beyond CBME, the findings help medical educators understand the impact of major changes to programs and curricula when done concurrently. These impacts can be mitigated with flexibility, clear communication, training, and identifying and addressing change fatigue [6, 7].

### Strengths and limitations

There are several strengths and limitations of our study. The strengths of the study are that the realist evaluation provided insight into the contexts and processes that result in outcomes that can be sometimes difficult to identify using other evaluation approaches. This methodology allowed us to identify novel issues that related to context that can have a profound impact on the implementation of educational innovations.

There are several limitations to the study. We discuss the impact of the concurrent implementation of a new CMS and CBME at a single institution, and therefore expect there to be variation in how different concurrent initiatives impact the implementation of CBME and other major curricular changes at different locations. Our decision to rely on qualitative data also limited our ability to test the degree to which rival initiatives impacted CBME outcomes and the strength of these associations.

## Conclusions

Our realist evaluation demonstrated that the UM’s implementation of CBME for specialty training programs was greatly affected by the implementation of a new CMS that was concurrent with, yet separate from CBME. Such decisions made at the institutional level in the academic health sciences context can have a significant impact on programs and individuals’ actions and reactions when implementing CBME and can ultimately affect the outcomes of CBME.

## Supplementary Information

**Additional file 1.**

## Data Availability

The datasets generated and analysed during the current study are not publicly available in order to maintain participant confidentiality but are available from the corresponding author on reasonable request.

## Abbreviations

CBD: Competence By Design
CBME: Competency-based medical education
CMO: Context + Mechanism = Outcome configuration
CMS: Curriculum management system
F: Faculty
PA: Program administrator
PD: Program director
PGME: Postgraduate medical education
R: Resident
UM: University of Manitoba
