# Supplementary material for: Rivalries for attention: insights from a realist evaluation of a postgraduate competency-based medical education implementation in Canada
Source: BMC Med Educ. 2022 Jul 29;22:583. doi: 10.1186/s12909-022-03661-8 (PMC9336173; doi:10.1186/s12909-022-03661-8)
Supplement: Supplementary file 1 — Additional file 1. [file 12909_2022_3661_MOESM1_ESM.docx]

**PHASE 1 INTERVIEW AND FOCUS GROUP GUIDES**

Interview Guide for Program Directors and Program Administrators

*Background*

1. How long have you been working for the college of medicine?

2. How long have you been in your current role?

3. What other roles have you held in the college of medicine or the university?

*Pre-Implementation*

4. Can you describe your role and experience in preparing your program for the launch of CBME?

PROBES: Can you describe your experience with:

- Communicating and receiving information about CBME?
- Accessing supports provided by PGME?
- Preparing faculty and support staff for CBME?
- Learning and establishing new systems and procedures?

5. What were your expectations for CBME before implementation began in your program?

PROBE: What were your expectations for:

For PDs:

- Frequency and variety of assessments?
- Making decisions about resident progress?
- Length of training?
- Your own workload?

For PAs:

- Using new technology?
- Communicating with and supporting residents and faculty?
- Your workload?

6. How, or from whom, did you learn about what to expect with CBME?

*Implementation*

7. Can you describe your experience with the implementation of CBME in your program?

PROBE: Describe the first few months of CBME in your program. What experiences stand out for you?

8. How has your experience compared to your expectations of CBME prior to implementation?

PROBE:

- How has your experience with CBME been similar to and/or different from what you expected?
- Why do you think your experience with CBME has been similar and/or different from your expectations?

8. What is the most significant change that CBME has brought to your program? Why is this change significant to you?

Focus Group Guide for Residents

1. What were your expectations for residency before you began your program?

PROBE: What were your expectations for:

- The role of faculty in your learning? For example, supervision or teaching?
- Your role in learning?
- Assessment?

2. How, or from whom, did you learn about what to expect during residency?

3. How has your experience of residency compared to your expectations?

PROBE: What was the same as expected? What was different from expected?

4. How have your expectations changed and what are your expectations for your residency now?

PROBE: What are your expectations now for:

- The role of faculty in your learning? For example, supervision or teaching?
- Your role in learning?
- Assessment?

5. To what degree does CBME contribute to any differences that may exist between your expectations for and experience of residency?

PROBE: How, if at all, has CBME changed your expectations regarding:

- The role of faculty in your learning? For example, supervision?
- Your role in learning?
- Assessment?

Focus Group Guide for Faculty

1. What were your expectations for CBME before implementation began in your program?

PROBE: What were your expectations for:

- Your role in resident learning?
- Residents’ roles in their learning?
- Teaching residents?
- Assessment of residents?
- Supervision of residents?

2. How, or from whom, did you learn about what to expect with CBME?

3. How have these expectations been met or not met?

PROBE:

- How has your experience with CBME been similar to and/or different from what you expected?
- Why do you think your experience with CBME has been similar and/or different from your expectations?

4. What is the most significant change that CBD has brought to your work?

PROBE: Why is this change significant to you?

**PHASE 2 INTERVIEW AND FOCUS GROUP GUIDES**

Interview Guide for Program Directors and Program Administrators

*Background*

1. How long have you been working for the college of medicine?

2. How long have you been in your current role?

3. What other roles have you held in the college of medicine or the university?

*Introductory*

4. Can you describe your role and experience with CBME?

PROBES: Can you describe your experience with:

- Communicating and receiving information about CBME?
- Accessing supports provided by PGME?
- Preparing faculty and support staff for CBME?
- Learning and establishing new systems and procedures?

5. When we spoke with other people in your role previously, they described their experience of CBD implementation as [summary of previous year’s findings]. In what ways is your experience of CBD the same or different? Why do you think this is so?

*Exploring Mechanisms*

6. [Follow-up interviews only] What, if any, changes have been made to the tasks and activities associated with CBME in your program in the past year?

PROBES: Can you describe any changes you’ve made to:

For PDS:

- The structure and process of the competence committee? Why did you make these changes?
- The CBME assessment tools and practices (e.g., EPAs)? Why did you make these changes?
- Curriculum or rotations? Why did you make these changes?

For PAs:

- How you track information about residents? What is the reason for this change?
- How you support the PD? What is the reason for this change?
- How you interact with faculty or residents? What is the reason for this change?

7. What is working well with the implementation of CBME in your program?

PROBES: Can you describe what is working well for:

- Your work/position? Why is it working well?
- Competence committees? Why is it working well?
- Teaching and learning? Why is it working well?

8. What, if any, difficulties have you encountered with the implementation of CBME?

PROBES: How have these difficulties affected:

- Your work?
- Residents?
- Faculty?
- Program Director or Administrator?

*Exploring Context*

9. What characteristics of your particular context affect how you carry out the tasks and activities associated with CBME?

PROBES: Describe any characteristics of the following that are a factor in CBME practices:

- Program Director (e.g., supportive)
- Faculty (e.g., no buy-in)
- Residents (e.g., highly motivated)
- Residency Program/Department (e.g., small program)
- PGME (e.g., clearly articulated guidelines and policies)
- Other external factors (e.g., changes in WRHA)

*Exploring Outcomes*

9. What is the most significant change that CBD has brought to your work?

PROBE: Why is this change significant to you?

10. What would you consider a successful implementation of CBD?

PROBES: Can you describe a desirable outcome for:

- You?
- Residents?
- Faculty?
- Society?

11. Based on your previous answers, to what degree do you think implementation has been successful?

Focus Group Guide for Residents

*Background*

1. Can you describe your experience with residency so far?

PROBES: Can you describe your experience with:

- - Learning about what to expect during residency?
  - Teaching, coaching, and the academic sessions in your program?
  - Being assessed and receiving feedback on your performance?
  - Learning how to use Entrada?
  - Mentoring and/or academic advising?

2. To what degree do you think CBD has had an impact on your experience of residency?

PROBES:

- - [For residents who started in CBD] How familiar are you with the difference between CBD and a traditional program?
  - [For residents who switched to CBD during residency] How has your experience of residency changed since you switched to CBD?
  - [For non-CBD residents] Has the launch of CBD in the cohorts below you affected your experience of residency?

3. When we spoke with residents last year about their experience with CBME, they described their experience as [summary of previous year’s findings]. In what ways is your experience of CBD the same or different? Why do you think this is so?

*Exploring Mechanisms*

4. [For CBME residents] What is working well with your residency?

PROBES: Can you describe what is working well for:

- - Achieving EPAs? Why is it working well?
  - Seeking assessments and receiving feedback? Why is it working well?
  - Your learning goals? Why is it working well?
  - Being coached and mentored? Why is it working well?

5. [For CBME residents] What, if any, difficulties have you encountered in your residency?

PROBES: How have these difficulties affected:

- - Your learning?
  - Your progress?

6. [For non-CBD residents] In what ways, if any, do you approach your learning differently since CBD launched in your program?

PROBES: Is there a difference in:

- - How you make learning goals? What is the reason for this change?
  - How you track your own progress? What is the reason for this change?
  - How you respond to feedback? What is the reason for this change?

*Exploring Context*

7. What characteristics of your particular context affect your training and learning in the program including, for example, your ability to achieve EPAs or receive coaching?

PROBES: Has your training or learning been affected by any characteristics of:

- - Your teachers or evaluators (e.g., tech savvy)
  - Training program (e.g., lots of off-service rotations)
  - PGME (e.g., clearly articulated guidelines and policies)
  - Other external factors (e.g., changes in WRHA)

*Exploring Outcomes*

8. What would you consider a successful implementation of CBD?

PROBES: Can you describe a desirable outcome for:

- - You?
  - The training program more broadly?
  - Society?

9. Based on your previous answers, to what degree do you think implementation has been successful?

**PHASE 3 INTERVIEW GUIDES**

Interview Guide for Program Directors and Program Administrators

*Background*

1. How long have you been working for the college of medicine?
2. How long have you been in your current role?
3. What other roles have you held in the college of medicine or the university?

*Theory Consolidation*

We have conducted interviews with program directors and program administrators and focus groups with residents and faculty members each spring starting in 2018. As we have collected and analyzed this data, we have begun to develop a tentative theory of what factors have influenced the implementation of CBME at the University of Manitoba. To begin our interview, I am going to show you a diagram that represents our tentative theory and I will walk you through it as well.

[Show participant diagram – provide summary of key findings represented in diagram]

4. How well does this theory reflect your experience with implementing CBME in your program?

PROBES:

- In what ways is your experience similar?
- In what ways is your experience different?
- Where do you see your program fitting within this theory?

5. Based on your experience, are there any significant experiences you have had with implementation that are not addressed in our theory?

PROBES:

- What made this experience significant?
- How did you or others react to this experience?
  - How did this significant experience affect:

1. you and your work?
2. the PD/PA and the PD/PA’s work?
3. residents?
4. teachers?
5. the program in general?

6. What would you consider a successful implementation of CBD?

PROBES: Can you describe a desirable outcome for:

- You?

- Residents?

- Teachers?

- Your program?

- Society?

7. Based on your previous answers, to what degree do you think implementation has been successful?

8. That is all the planned questions I have, before we finish the interview, is there anything else you would like to add about your experience with implementing CBD that we haven’t already discussed?
